# 2-(6-Meth­oxy­naphthalen-2-yl)-1-(morpholin-4-yl)propan-1-one

**DOI:** 10.1107/S1600536812034083

**Published:** 2012-08-04

**Authors:** Nasirullah, Nazar Ul Islam, M. Nawaz Tahir, Ikhtiar Khan, Muhammad Zulfiqar

**Affiliations:** a Institute of Chemical Sciences, University of Peshawar, Peshawar, Pakistan; b University of Sargodha, Department of Physics, Sargodha, Pakistan

## Abstract

In the title compound, C_18_H_21_NO_3_, the naphthalene group and the basal plane of the morpholine ring (r.m.s. deviations = 0.0177 and 0.0069 Å, respectively) are oriented at a dihedral angle of 44.0 (2)°. In the crystal, mol­ecules are linked by C—H⋯π inter­actions.

## Related literature

For the crystal structure of the related compound, naproxen [systematic name: (+)-2-(6-meth­oxy-2-naphth­yl)-propionic acid], see: Ravikumar *et al.* (1985 ▶).
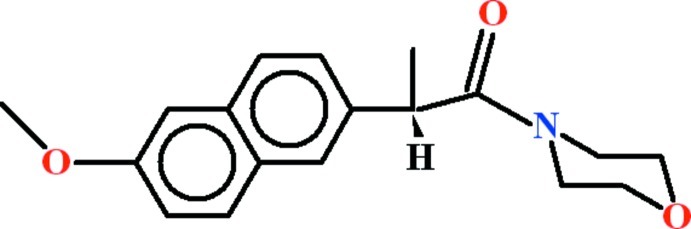


## Experimental

### 

#### Crystal data

C_18_H_21_NO_3_
*M* *_r_* = 299.36
Monoclinic, 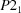

*a* = 9.5947 (15) Å
*b* = 6.6293 (8) Å
*c* = 12.340 (2) Å
β = 92.221 (5)°
*V* = 784.3 (2) Å^3^
*Z* = 2
Mo *K*α radiation
μ = 0.09 mm^−1^
*T* = 296 K
0.33 × 0.23 × 0.17 mm

#### Data collection

Bruker Kappa APEXII CCD diffractometer
Absorption correction: multi-scan (*SADABS*; Bruker, 2005 ▶) *T*_min_ = 0.972, *T*_max_ = 0.986
6522 measured reflections
1681 independent reflections
1029 reflections with *I* > 2σ(*I*)
*R*_int_ = 0.047

#### Refinement

*R*[*F* ^2^ > 2σ(*F* ^2^)] = 0.054
*wR*(*F* ^2^) = 0.142
*S* = 1.02
1681 reflections
201 parameters
1 restraint
H-atom parameters constrained
Δρ_max_ = 0.18 e Å^−3^
Δρ_min_ = −0.19 e Å^−3^

### 

Data collection: *APEX2* (Bruker, 2009 ▶); cell refinement: *SAINT* (Bruker, 2009 ▶); data reduction: *SAINT*; program(s) used to solve structure: *SHELXS97* (Sheldrick, 2008 ▶); program(s) used to refine structure: *SHELXL97* (Sheldrick, 2008 ▶); molecular graphics: *ORTEP-3 for Windows* (Farrugia, 1997 ▶) and *PLATON* (Spek, 2009 ▶); software used to prepare material for publication: *WinGX* (Farrugia, 1999 ▶) and *PLATON*.

## Supplementary Material

Crystal structure: contains datablock(s) global, I. DOI: 10.1107/S1600536812034083/su2485sup1.cif

Structure factors: contains datablock(s) I. DOI: 10.1107/S1600536812034083/su2485Isup2.hkl

Supplementary material file. DOI: 10.1107/S1600536812034083/su2485Isup3.cml

Additional supplementary materials:  crystallographic information; 3D view; checkCIF report

## Figures and Tables

**Table 1:** Hydrogen-bond geometry (Å, °) *Cg*2 and *Cg*3 are the centroids of the C1–C6 and C3/C4/C7–C10 rings, respectively.

*D*—H⋯*A*	*D*—H	H⋯*A*	*D*⋯*A*	*D*—H⋯*A*
C7—H7⋯*Cg*3^i^	0.93	2.98	3.679 (5)	133
C15—H15*A*⋯*Cg*3^ii^	0.97	2.95	3.756 (5)	141
C16—H16*A*⋯*Cg*2^ii^	0.97	2.79	3.675 (5)	153

Symmetry codes: (i) 
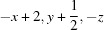
; (ii) 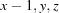.

**Fig. 1.**
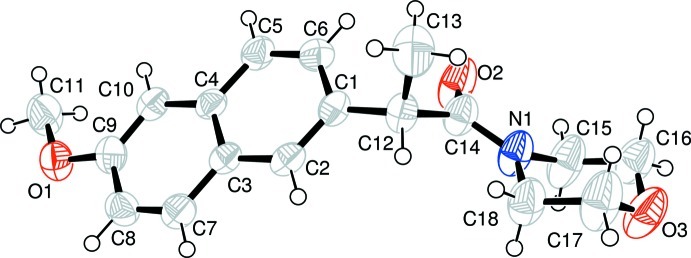
A view of the molecular structure of the title molecule, with atom numbering. Displacement ellipsoids are drawn at the 50% probability level.

## supplementary crystallographic information

### Comment

The title compound is the morpholine derivative of Naproxen [(+)-2-(6-methoxy-2-naphthyl)-propionic acid], whose crystal structure has been reported on by (Ravikumar *et al.*, 1985). The title compound was synthesized in order to study its biological properties and we report herein on its synthesis and crystal structure.

The molecular structure of the title compound is illustrated in Fig. 1. The naphthaline group A (C1–C10) and the basal plane of the morpholine group B (atoms C15—C18) are planar with r.m.s. deviations of 0.0177 Å and 0.0069 Å, respectively. The dihedral angle between planes A/B is 43.97 (23)°. The O1 and C11 atoms of the methoxy group are at a distance of -0.0911 (44) and -0.2335 (74) Å, respectively, from the mean plane of the naphthaline group. The morpholine group has a chair conformation with atoms N1 and O3 at a distance of 0.5827 (79) and -0.6752 (77) Å, respectively, from the basal plane B.

In the crystal, molecules are linked via C—H···π interactions (Table 1).

### Experimental

A solution of morpholine (0.35 g, 40.2 mmol) in 5 ml of dichloromethane (DCM) was added to a solution of naproxen acid chloride (0.5 g, 20.1 mmol) in DCM (10 ml). The reaction mixture was stirred at room temperature for 3 h. After completion the reaction mixture was filtered and the filtrate concentrated to give the crude product. The product was purified by flash column chromatogrphy using n-hexane: ethyl acetate (50:50). The resulting jelly like product was recystallized from diethyl ether and hexane (1:1) to give the title compound as colourless prism-like crystals, suitable for X-ray diffraction analysis [Yield: 65.0%, M.p.: 388 K].

### Refinement

In the final cycles of refinement, in the absence of significant anomalous scattering effects, Friedel pairs were merged and Δf " set to zero. The H atoms were positioned geometrically (C–H = 0.93–0.98 Å) and refined as riding with *U*_iso_(H) = k × U_eq_(C), where *k* = 1.5 for methyl and = 1.2 for other H-atoms.

### Crystal data

**Table:**

C_18_H_21_NO_3_	*F*(000) = 320
*M**_r_* = 299.36	*D*_x_ = 1.268 Mg m^−^^3^
Monoclinic, *P*2_1_	Mo *K*α radiation, λ = 0.71073 Å
Hall symbol: P 2yb	Cell parameters from 1029 reflections
*a* = 9.5947 (15) Å	θ = 1.7–26.0°
*b* = 6.6293 (8) Å	µ = 0.09 mm^−^^1^
*c* = 12.340 (2) Å	*T* = 296 K
β = 92.221 (5)°	Prism, colourless
*V* = 784.3 (2) Å^3^	0.33 × 0.23 × 0.17 mm
*Z* = 2	

### Data collection

**Table:**

Bruker Kappa APEXII CCD diffractometer	1681 independent reflections
Radiation source: fine-focus sealed tube	1029 reflections with *I* > 2σ(*I*)
Graphite monochromator	*R*_int_ = 0.047
Detector resolution: 8.00 pixels mm^-1^	θ_max_ = 26.0°, θ_min_ = 1.7°
ω scans	*h* = −11→11
Absorption correction: multi-scan (*SADABS*; Bruker, 2005)	*k* = −8→7
*T*_min_ = 0.972, *T*_max_ = 0.986	*l* = −15→15
6522 measured reflections	

### Refinement

**Table:**

Refinement on *F*^2^	Primary atom site location: structure-invariant direct methods
Least-squares matrix: full	Secondary atom site location: difference Fourier map
*R*[*F*^2^ > 2σ(*F*^2^)] = 0.054	Hydrogen site location: inferred from neighbouring sites
*wR*(*F*^2^) = 0.142	H-atom parameters constrained
*S* = 1.02	*w* = 1/[σ^2^(*F*_o_^2^) + (0.0707*P*)^2^] where *P* = (*F*_o_^2^ + 2*F*_c_^2^)/3
1681 reflections	(Δ/σ)_max_ < 0.001
201 parameters	Δρ_max_ = 0.18 e Å^−^^3^
1 restraint	Δρ_min_ = −0.19 e Å^−^^3^

### Special details

**Table:**

Geometry. Bond distances, angles *etc*. have been calculated using the rounded fractional coordinates. All su's are estimated from the variances of the (full) variance-covariance matrix. The cell e.s.d.'s are taken into account in the estimation of distances, angles and torsion angles
Refinement. Refinement of *F*^2^ against ALL reflections. The weighted *R*-factor *wR* and goodness of fit *S* are based on *F*^2^, conventional *R*-factors *R* are based on *F*, with *F* set to zero for negative *F*^2^. The threshold expression of *F*^2^ > σ(*F*^2^) is used only for calculating *R*-factors(gt) *etc*. and is not relevant to the choice of reflections for refinement. *R*-factors based on *F*^2^ are statistically about twice as large as those based on *F*, and *R*- factors based on ALL data will be even larger.

### Fractional atomic coordinates and isotropic or equivalent isotropic displacement parameters (Å^2^)

**Table:**

	*x*	*y*	*z*	*U*_iso_*/*U*_eq_	
O1	1.3551 (3)	0.2256 (4)	−0.0544 (3)	0.0602 (11)	
O2	0.6416 (3)	0.3801 (5)	0.3319 (3)	0.0829 (16)	
O3	0.2888 (3)	0.8958 (5)	0.3399 (3)	0.0884 (14)	
N1	0.5373 (4)	0.6801 (5)	0.3199 (3)	0.0609 (14)	
C1	0.9024 (4)	0.5452 (6)	0.2733 (3)	0.0465 (16)	
C2	0.9648 (4)	0.6297 (6)	0.1868 (3)	0.0460 (14)	
C3	1.0673 (4)	0.5277 (6)	0.1281 (3)	0.0425 (14)	
C4	1.1077 (4)	0.3316 (6)	0.1603 (3)	0.0436 (16)	
C5	1.0438 (4)	0.2470 (6)	0.2511 (4)	0.0513 (14)	
C6	0.9445 (4)	0.3484 (6)	0.3041 (4)	0.0530 (17)	
C7	1.1283 (4)	0.6141 (7)	0.0367 (4)	0.0511 (14)	
C8	1.2234 (4)	0.5100 (7)	−0.0192 (3)	0.0533 (17)	
C9	1.2619 (4)	0.3142 (7)	0.0120 (4)	0.0496 (16)	
C10	1.2068 (4)	0.2259 (6)	0.1004 (3)	0.0482 (14)	
C11	1.3915 (5)	0.0220 (7)	−0.0360 (5)	0.081 (2)	
C12	0.7953 (4)	0.6652 (7)	0.3314 (4)	0.0522 (16)	
C13	0.8407 (5)	0.7042 (9)	0.4502 (4)	0.079 (2)	
C14	0.6520 (4)	0.5624 (7)	0.3276 (4)	0.0573 (19)	
C15	0.3990 (4)	0.5883 (8)	0.3042 (5)	0.083 (2)	
C16	0.2948 (5)	0.6889 (8)	0.3671 (5)	0.074 (2)	
C17	0.4198 (5)	0.9877 (8)	0.3664 (6)	0.093 (3)	
C18	0.5324 (5)	0.8992 (7)	0.3048 (5)	0.076 (2)	
H2	0.93901	0.75949	0.16555	0.0550*	
H5	1.07044	0.11908	0.27494	0.0613*	
H6	0.90287	0.28688	0.36230	0.0634*	
H7	1.10287	0.74354	0.01461	0.0612*	
H8	1.26357	0.56916	−0.07875	0.0638*	
H10	1.23423	0.09657	0.12116	0.0575*	
H11A	1.43560	0.00846	0.03477	0.1220*	
H11B	1.45462	−0.02119	−0.08983	0.1220*	
H11C	1.30890	−0.05985	−0.04048	0.1220*	
H12	0.78519	0.79615	0.29504	0.0627*	
H13A	0.84608	0.57838	0.48862	0.1191*	
H13B	0.77382	0.79042	0.48299	0.1191*	
H13C	0.93047	0.76826	0.45328	0.1191*	
H15A	0.37086	0.59422	0.22793	0.0991*	
H15B	0.40414	0.44741	0.32518	0.0991*	
H16A	0.20434	0.62728	0.35244	0.0888*	
H16B	0.31828	0.67402	0.44387	0.0888*	
H17A	0.44130	0.97154	0.44335	0.1111*	
H17B	0.41381	1.13106	0.35108	0.1111*	
H18A	0.51725	0.92988	0.22838	0.0903*	
H18B	0.62088	0.95783	0.32891	0.0903*	

### Atomic displacement parameters (Å^2^)

**Table:**

	*U*^11^	*U*^22^	*U*^33^	*U*^12^	*U*^13^	*U*^23^
O1	0.0555 (18)	0.053 (2)	0.073 (2)	0.0001 (15)	0.0157 (16)	−0.0012 (16)
O2	0.052 (2)	0.039 (2)	0.158 (4)	0.0020 (15)	0.009 (2)	0.000 (2)
O3	0.062 (2)	0.056 (2)	0.147 (3)	0.0147 (17)	0.002 (2)	0.007 (2)
N1	0.044 (2)	0.037 (2)	0.102 (3)	0.0007 (17)	0.007 (2)	−0.0035 (19)
C1	0.036 (2)	0.050 (3)	0.053 (3)	0.000 (2)	−0.004 (2)	−0.003 (2)
C2	0.040 (2)	0.037 (2)	0.060 (3)	−0.0007 (19)	−0.010 (2)	0.003 (2)
C3	0.037 (2)	0.042 (2)	0.048 (3)	0.0008 (19)	−0.0062 (19)	0.0000 (19)
C4	0.036 (2)	0.046 (3)	0.048 (3)	−0.0011 (18)	−0.007 (2)	0.004 (2)
C5	0.048 (2)	0.038 (2)	0.068 (3)	0.0066 (19)	0.003 (2)	0.008 (2)
C6	0.054 (3)	0.050 (3)	0.055 (3)	0.004 (2)	0.001 (2)	0.010 (2)
C7	0.046 (2)	0.040 (2)	0.067 (3)	−0.004 (2)	−0.003 (2)	0.007 (2)
C8	0.052 (3)	0.054 (3)	0.054 (3)	−0.009 (2)	0.004 (2)	0.006 (2)
C9	0.044 (2)	0.049 (3)	0.056 (3)	−0.006 (2)	0.003 (2)	−0.009 (2)
C10	0.042 (2)	0.044 (2)	0.058 (3)	0.0044 (19)	−0.004 (2)	0.001 (2)
C11	0.079 (4)	0.063 (4)	0.104 (4)	0.026 (3)	0.037 (3)	0.016 (3)
C12	0.042 (2)	0.049 (3)	0.066 (3)	0.002 (2)	0.007 (2)	−0.004 (2)
C13	0.064 (3)	0.099 (4)	0.075 (4)	−0.007 (3)	0.000 (3)	−0.029 (3)
C14	0.048 (3)	0.043 (3)	0.081 (4)	0.001 (2)	0.004 (2)	−0.003 (2)
C15	0.044 (3)	0.060 (4)	0.144 (5)	−0.004 (2)	0.002 (3)	−0.012 (3)
C16	0.046 (3)	0.065 (4)	0.111 (4)	−0.001 (3)	−0.001 (3)	0.003 (3)
C17	0.067 (4)	0.048 (3)	0.165 (6)	0.000 (3)	0.023 (4)	−0.010 (4)
C18	0.062 (3)	0.047 (3)	0.119 (5)	0.007 (2)	0.023 (3)	0.012 (3)

### Geometric parameters (Å, º)

**Table:**

O1—C9	1.369 (5)	C17—C18	1.467 (8)
O1—C11	1.410 (5)	C2—H2	0.9300
O2—C14	1.214 (6)	C5—H5	0.9300
O3—C16	1.413 (6)	C6—H6	0.9300
O3—C17	1.423 (6)	C7—H7	0.9300
N1—C14	1.349 (6)	C8—H8	0.9300
N1—C15	1.466 (6)	C10—H10	0.9300
N1—C18	1.465 (6)	C11—H11A	0.9600
C1—C2	1.364 (5)	C11—H11B	0.9600
C1—C6	1.413 (6)	C11—H11C	0.9600
C1—C12	1.503 (6)	C12—H12	0.9800
C2—C3	1.416 (5)	C13—H13A	0.9600
C3—C4	1.409 (6)	C13—H13B	0.9600
C3—C7	1.412 (6)	C13—H13C	0.9600
C4—C5	1.414 (6)	C15—H15A	0.9700
C4—C10	1.413 (5)	C15—H15B	0.9700
C5—C6	1.355 (6)	C16—H16A	0.9700
C7—C8	1.354 (6)	C16—H16B	0.9700
C8—C9	1.400 (6)	C17—H17A	0.9700
C9—C10	1.363 (6)	C17—H17B	0.9700
C12—C13	1.535 (7)	C18—H18A	0.9700
C12—C14	1.534 (6)	C18—H18B	0.9700
C15—C16	1.452 (7)		
			
C9—O1—C11	118.5 (4)	C8—C7—H7	120.00
C16—O3—C17	109.5 (4)	C7—C8—H8	120.00
C14—N1—C15	120.1 (4)	C9—C8—H8	120.00
C14—N1—C18	127.2 (4)	C4—C10—H10	120.00
C15—N1—C18	111.7 (4)	C9—C10—H10	120.00
C2—C1—C6	117.4 (4)	O1—C11—H11A	109.00
C2—C1—C12	119.0 (4)	O1—C11—H11B	109.00
C6—C1—C12	123.6 (4)	O1—C11—H11C	109.00
C1—C2—C3	122.6 (4)	H11A—C11—H11B	110.00
C2—C3—C4	119.0 (3)	H11A—C11—H11C	109.00
C2—C3—C7	122.2 (4)	H11B—C11—H11C	109.00
C4—C3—C7	118.8 (4)	C1—C12—H12	108.00
C3—C4—C5	117.8 (3)	C13—C12—H12	108.00
C3—C4—C10	119.6 (3)	C14—C12—H12	108.00
C5—C4—C10	122.6 (4)	C12—C13—H13A	109.00
C4—C5—C6	121.4 (4)	C12—C13—H13B	109.00
C1—C6—C5	121.8 (4)	C12—C13—H13C	109.00
C3—C7—C8	120.6 (4)	H13A—C13—H13B	109.00
C7—C8—C9	120.6 (4)	H13A—C13—H13C	110.00
O1—C9—C8	113.9 (4)	H13B—C13—H13C	109.00
O1—C9—C10	125.3 (4)	N1—C15—H15A	109.00
C8—C9—C10	120.8 (4)	N1—C15—H15B	109.00
C4—C10—C9	119.7 (4)	C16—C15—H15A	109.00
C1—C12—C13	111.8 (4)	C16—C15—H15B	109.00
C1—C12—C14	112.3 (4)	H15A—C15—H15B	108.00
C13—C12—C14	109.0 (4)	O3—C16—H16A	110.00
O2—C14—N1	120.7 (4)	O3—C16—H16B	110.00
O2—C14—C12	121.1 (4)	C15—C16—H16A	110.00
N1—C14—C12	118.2 (4)	C15—C16—H16B	110.00
N1—C15—C16	112.2 (4)	H16A—C16—H16B	108.00
O3—C16—C15	110.0 (4)	O3—C17—H17A	109.00
O3—C17—C18	111.8 (5)	O3—C17—H17B	109.00
N1—C18—C17	110.6 (4)	C18—C17—H17A	109.00
C1—C2—H2	119.00	C18—C17—H17B	109.00
C3—C2—H2	119.00	H17A—C17—H17B	108.00
C4—C5—H5	119.00	N1—C18—H18A	110.00
C6—C5—H5	119.00	N1—C18—H18B	110.00
C1—C6—H6	119.00	C17—C18—H18A	110.00
C5—C6—H6	119.00	C17—C18—H18B	110.00
C3—C7—H7	120.00	H18A—C18—H18B	108.00
			
C11—O1—C9—C10	4.6 (6)	C4—C3—C7—C8	0.3 (6)
C11—O1—C9—C8	−174.5 (4)	C7—C3—C4—C10	−0.5 (6)
C17—O3—C16—C15	−62.1 (6)	C2—C3—C7—C8	−178.3 (4)
C16—O3—C17—C18	61.9 (6)	C7—C3—C4—C5	−179.1 (4)
C18—N1—C15—C16	−50.5 (6)	C2—C3—C4—C5	−0.5 (6)
C14—N1—C15—C16	140.2 (5)	C2—C3—C4—C10	178.1 (4)
C18—N1—C14—O2	−174.4 (5)	C3—C4—C5—C6	1.6 (6)
C18—N1—C14—C12	5.9 (7)	C3—C4—C10—C9	−0.2 (6)
C15—N1—C14—O2	−7.0 (7)	C5—C4—C10—C9	178.3 (4)
C15—N1—C18—C17	48.5 (6)	C10—C4—C5—C6	−177.0 (4)
C14—N1—C18—C17	−143.2 (5)	C4—C5—C6—C1	−1.7 (7)
C15—N1—C14—C12	173.4 (4)	C3—C7—C8—C9	0.6 (6)
C6—C1—C12—C14	−61.7 (5)	C7—C8—C9—C10	−1.4 (6)
C6—C1—C2—C3	0.4 (6)	C7—C8—C9—O1	177.8 (4)
C2—C1—C12—C13	−118.0 (4)	O1—C9—C10—C4	−177.9 (4)
C6—C1—C12—C13	61.1 (5)	C8—C9—C10—C4	1.1 (6)
C12—C1—C6—C5	−178.4 (4)	C13—C12—C14—N1	91.2 (5)
C2—C1—C12—C14	119.2 (4)	C13—C12—C14—O2	−88.5 (6)
C2—C1—C6—C5	0.7 (6)	C1—C12—C14—N1	−144.5 (4)
C12—C1—C2—C3	179.6 (4)	C1—C12—C14—O2	35.8 (6)
C1—C2—C3—C7	178.1 (4)	N1—C15—C16—O3	57.1 (6)
C1—C2—C3—C4	−0.5 (6)	O3—C17—C18—N1	−54.8 (6)

### Hydrogen-bond geometry (Å, º)

Cg2 and Cg3 are the centroids of the C1–C6 and C3/C4/C7–C10 rings, respectively.

**Table:**

*D*—H···*A*	*D*—H	H···*A*	*D*···*A*	*D*—H···*A*
C7—H7···*Cg*3^i^	0.93	2.98	3.679 (5)	133
C15—H15*A*···*Cg*3^ii^	0.97	2.95	3.756 (5)	141
C16—H16*A*···*Cg*2^ii^	0.97	2.79	3.675 (5)	153

Symmetry codes: (i) −*x*+2, *y*+1/2, −*z*; (ii) *x*−1, *y*, *z*.
